# Swellable and Thermally Responsive Hydrogel/Shape Memory Polymer Foam Composites for Sealing Lung Biopsy Tracts

**DOI:** 10.1021/acsbiomaterials.2c01369

**Published:** 2023-02-02

**Authors:** Matthew A. Jungmann, Sarea Recalde Phillips, Tyler J. Touchet, Braeden Brinson, Katherine Parish, Corinne Petersen, Sayyeda Marziya Hasan, Landon D. Nash, Duncan J. Maitland, Daniel L. Alge

**Affiliations:** † Department of Biomedical Engineering, Texas A&M University, College Station, Texas 77843, United States; ‡ Department of Chemical Engineering, Texas A&M University, College Station, Texas 77843, United States; § Shape Memory Medical, Inc., Santa Clara, California 95054, United States; ∥ Department of Materials Science & Engineering, Texas A&M University, College Station, Texas 77843, United States

**Keywords:** PEG hydrogel, shape memory polymer foam, composite, lung biopsy sealant

## Abstract

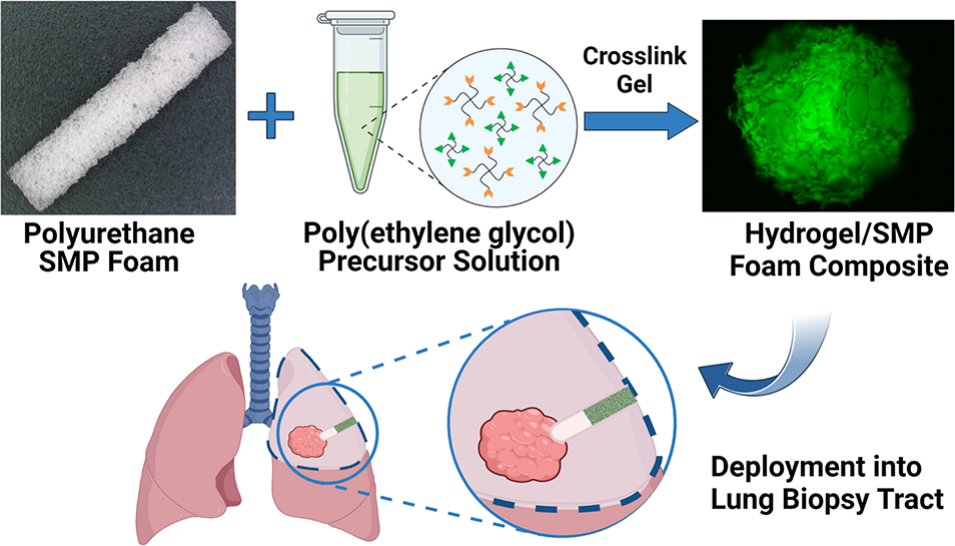

Lung tissue biopsies can result in a leakage of blood (hemothorax) and air (pneumothorax) from the biopsy tract, which threatens the patient with a collapsed lung and other complications. We have developed a lung biopsy tract sealant based on a thiol–ene-crosslinked PEG hydrogel and polyurethane shape memory polymer (SMP) foam composite. After insertion into biopsy tracts, the PEG hydrogel component contributes to sealing through water-driven swelling, whereas the SMP foam contributes to sealing via thermal actuation. The gelation kinetics, swelling properties, and rheological properties of various hydrogel formulations were studied to determine the optimal formulation for composite fabrication. Composites were then fabricated via vacuum infiltration of the PEG hydrogel precursors into the SMP foam followed by thermal curing. After drying, the composites were crimped to enable insertion into biopsy tracts. Characterization revealed that the composites exhibited a slight delay in shape recovery compared to control SMP foams. However, the composites were still able to recover their shape in a matter of minutes. Cytocompatibility testing showed that leachable byproducts can be easily removed by washing and washed composites were not cytotoxic to mouse lung fibroblasts (L929s). Benchtop testing demonstrated that the composites can be easily deployed through a cannula, and the working time for deployment after exposure to water was 2 min. Furthermore, testing in an in vitro lung model demonstrated that the composites were able to effectively seal a lung biopsy tract and prevent air leakage. Collectively, these results show that the PEG hydrogel/SMP foam composites have the potential to be used as lung biopsy tract sealants to prevent pneumothorax post-lung biopsy.

## Introduction

Tissue biopsies are the current gold standard for definitively diagnosing multiple cancer types. In 2008, 677,811 tissue biopsies were performed in the United States, growing at a rate of ∼3% annually since 1997.^1^ Of these 677,000 tissue biopsies, around 101,000 were lung biopsies.^1^ If this rate continues as expected, it can be estimated that 1,025,000 biopsy procedures will be performed in the USA in 2022, with approximately 152,000 lung biopsies. Lung biopsies are performed percutaneously using a coaxial needle, a cannula, and guidance equipment for visualization. Post-procedural complications can arise from a lung biopsy due to the potential for leakage of air (pneumothorax) and blood (hemothorax) from the biopsy tract.^2^ Hemothorax is rare, occurring in about 0.1% cases in one study.^3^ However, pneumothorax is more prominent with 4.3–52.4% of lung biopsy procedures resulting in pneumothorax, and as many as 15% resulting in pneumothorax requiring chest tube placement, according to one meta-analysis.^4^

Clinicians often utilize unique techniques to seal and plug biopsy tracts. For example, an early developed technique was the use of autologous blood clots. These clots were proven effective in promoting healing and hemostasis but were susceptible to air leakage.^5,6^ The off-label use of fibrin-based tissue sealants (fibrin glue) and Gelfoam—a surgical hemostat made from a purified porcine extracellular matrix—has also been studied for use in lung biopsy tract sealing and prevention of pneumothorax.^7,8^ Although these studies do show a slight reduction in the incidence of pneumothorax, the use of fibrin glue and Gelfoam has not gained wide acceptance. More recently, the BioSentry Lung Biopsy Tract Plug System, an FDA-approved absorbable poly(ethylene glycol) (PEG) hydrogel plug, has been developed for sealing lung biopsy tracts to prevent the risk of pneumothoraxes.^9,10^ Briefly, the system consists of a PEG hydrogel plug and delivery device. After the tissue sample is collected, the PEG hydrogel plug is immersed in saline solution within the delivery device housing to pre-swell the plug. The plug is then deployed into the tract where it will swell in the extracellular fluid and expand to seal the tract, preventing leakage of air. One study has shown that patients that received the BioSentry device had significantly lower rates of pneumothorax (17.6 vs 30.2%) and chest tube placement (7.2 vs 18%) compared to those who did not.^11^ However, Grage et al. found that a BioSentry-treated group and control group (an unsealed biopsy tract) had similar pneumothorax rates, 30 and 31%, respectively.^12^ Furthermore, according to the BioSentry instructions for use, the interventional radiologist has just 30 s to deploy the hydrogel plug into the tract. This working time requires the radiologist to rush the placement of the hydrogel plug, increasing the chance of error in placement and, thus, the incidence of pneumothorax. Therefore, there is a need for new lung biopsy tract sealants with longer working times for simpler, more effective sealing of the tract and reduction of pneumothorax rates post-lung biopsy.

To meet this need, we are developing a lung biopsy sealant based on a thiol–ene click-crosslinked PEG hydrogel and polyurethane-based shape memory polymer (SMP) foam composite. The SMP foam serves as the base and has a dry PEG hydrogel coating throughout the pores of the foam. The two components of the composite play distinctly different roles in contributing to biopsy tract sealing. Because PEG is inherently hygroscopic, the dry PEG hydrogel coating is expected to quickly swell within the tract.^13−15^ On the other hand, the polyurethane SMP foam is a thermally responsive smart material that can transition between a deformed shape and original shape in response to a stimulus (e.g., heat).^16−18^ Leveraging the excellent shape fixity of the polyurethane SMP foam, the composites will be crimped down into a temporary shape and delivered into the lung biopsy tract.^17^ Once in the tract, the SMP foam will actuate and return to its original shape due to the body temperature and the plasticization of the polymer chains via interaction with extracellular fluid in the biopsy tract.^19^ The combination of the expanding SMP foam and swelling of the PEG hydrogel through fluid uptake will seal the biopsy tract and reduce the risk of pneumothorax. Because it is intended to be delivered dry, the composite will have a much longer working time compared to the BioSentry to ensure perfect placement.

The objective of this study was to develop and characterize our PEG hydrogel/SMP foam composites and demonstrate their ability to serve as a lung biopsy sealant through in vitro testing. Development of the composite began with studying the effects of PEG hydrogel formulation on properties relevant to composite fabrication and performance. Specifically, bulk PEG hydrogels with varying PEG weight % (wt %) and thiol to alkene (thiol–ene) molar ratios were evaluated based on their gelation kinetics, swelling properties, and rheological properties. A formulation was chosen based on these results, and composites were fabricated via vacuum infiltration of PEG hydrogel precursors into SMP foams and thermal curing. The hydrogel infiltration, shape recovery, and cytocompatibility of the composites were characterized to determine the effectiveness of the fabrication technique and to ensure that the addition of the hydrogel had no negative effects on the properties of the SMP foam. Finally, benchtop testing on the composites was performed to assess their ability to effectively be deployed and seal lung biopsy tracts.

## Experimental Section

### Materials

Four-arm PEG (MW = 20 kDa) (PEG-OH) and four-arm poly(ethylene glycol)-thiol (MW = 10 kDa) (PEG-SH) were purchased from JenKem Technology USA (Plano, Texas). Diisopropylcarbodiimide, 4-(dimethylamino)pyridine, 5-norbornene-2-carboxylic acid, and anhydrous dichloromethane were purchased from Sigma-Aldrich (St. Louis, Missouri). 2,2′-Azobis[2-(2-imidazolin-2-yl) propane]dihydrochloride (VA-044) was purchased from FUJIFILM Wako Pure Chemical Corporation (Richmond, Virginia). 5-((2-(and-3)-S-(Acetylmercapto) succinoyl) amino) (SAMSA) fluorescein was purchased from Fisher Scientific (Eugene, Oregon). Minimum Essential Medium, penicillin–streptomycin, and GlutaMAX were purchased from Fisher Scientific (Eugene, Oregon). SMP foams were graciously provided by Shape Memory Medical, Inc. (Santa Clara, California).

### Hydrogel Synthesis and Characterization

#### Synthesis of PEG-Tetra Norbornene (PEG-NB)

Four-arm PEG-NB was synthesized via esterification, according to an established protocol.^20^ Briefly, 10 g of PEG-OH (20 kDa, 4-arm), 4-(dimethylamino)pyridine (0.5 equiv to OH groups), and pyridine (10 equiv to OH groups) were dissolved in a round-bottom flask in anhydrous dichloromethane (40 mL) under argon gas. Meanwhile, 5-norbornene-2-carboxylic acid (10 COOH equiv to OH groups), diisopropylcarbodiimide (5 equiv to OH groups), and anhydrous dichloromethane were mixed in a reaction vessel for 45 min to form dinorbornene anhydride. The mixture from the reaction vessel was filtered into the PEG-OH-containing round-bottom flask, removing urea salt byproducts, and the reaction was allowed to proceed at room temperature overnight. The solution was precipitated in 1000 mL of chilled diethyl ether and vacuum filtered to collect the white precipitate (PEG-NB), which was then dried in a vacuum desiccator for 48 h. Finally, PEG-NB was dissolved in deionized water, added to dialysis tubing (MWCO = 10 kDa), and dialyzed against deionized water for 2 days, changing the water after 1 day. The final product was lyophilized and analyzed via ^1^H NMR to determine percent norbornene end-group functionalization of PEG (Figure S1). The percent functionalization was 93.5%.

#### Hydrogel Synthesis

Hydrogel disks (8 mm-diameter and 2 mm-thick) were synthesized via radical-mediated thiol–ene click crosslinking of four-arm PEG-NB (20 kDa) and four-arm PEG-SH (10 kDa).^21,22^ Briefly, hydrogel precursor solutions of varying PEG wt % (8, 12, and 16 wt %) and thiol-to-alkene ratios (1.5, 2.0, and 2.5) were made with PEG-NB, PEG-SH, and the thermal azo initiator VA-044 (0.5 wt %). The solutions were added into a silicone mold with 8 mm-diameter and 2 mm-thick cylindrical cutouts and fixed between two glass slides. The mold was subsequently placed into an oven at 65 °C for 30 min to form the hydrogels.

#### Gelation Kinetics

Thermal gelation kinetics were studied using a rheometer (MCR 301, Anton Paar) with a PP08 parallel plate. Hydrogel precursor solutions (*n* = 3) with varying PEG wt % (8, 12, 16 wt %) and thiol–ene ratios (1.5, 2.0, 2.5) were pipetted onto the rheometer stage at a volume of 100 μL. The plate was lowered until the entire solution covered the plate and the test was started. Time sweeps within the linear viscoelastic regime—at a frequency of 1 rad/s and 1% strain—were conducted at 65 °C. The time point at which the storage modulus (*G*′) increased sharply to exceed the loss modulus (*G*″), indicating the transition from solution to gel, was taken as the gelation time.

#### Equilibrium Swelling Ratio Measurements

Hydrogel disks (*n* = 3) were synthesized and desiccated in a vacuum desiccator overnight. The dry weights of the disks were recorded, and the disks were immersed in PBS for 17 h. The weights at equilibrium swelling were measured, and the equilibrium swelling ratio was calculated according to eq 1:
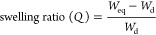
1where *W*_d_ and *W*_eq_ represent the initial dry weight and weight at equilibrium swelling, respectively.

#### Equilibrium Storage Modulus Measurements

Hydrogel disks (*n* = 3) were synthesized and swelled in PBS for 17 h, as described above. 8 mm disks were punched out of the swollen hydrogels with an 8 mm biopsy punch. The viscoelastic properties of the hydrogel disks were measured with a rheometer and a PP08 parallel plate. Frequency sweeps (0.1 to 10 rad/s at 1% shear strain) and amplitude sweeps (0.01 to 10% shear strain at 1 rad/s) were conducted on the 2.5 thiol–ene groups to determine the linear viscoelastic region. Thirty-second time sweeps at a frequency of 1 rad/s and 1% shear strain were subsequently conducted on the remaining groups. The storage modulus of each group was calculated as the average storage modulus over the 30 s of measurement at a frequency of 1 rad/s and 1% shear strain.

#### Swelling Kinetics

Hydrogel samples (*n* = 3) were desiccated or lyophilized. To lyophilize, samples were frozen in −80 °C overnight and then placed on a lyophilizer (Labconco FreeZone 4.5 Plus) at 0.01 mbar and −84 °C for 24 h. Before testing, the dry weight of each sample was recorded. The samples were immersed in PBS and removed, dried, and weighed at various timepoints. The mass of PBS absorbed at a timepoint was calculated as the weight of the hydrogel when removed from the PBS minus the dry weight. The timepoints considered were 1, 2, 3, 4, 5, 10, 15, 30, 60, 120, 180, 240, 300, and 1020 min (equilibrium). Swelling rates were determined by fitting the first five measurements to a linear trendline, where the slope of the trendline was the rate of swelling.

### Composite Fabrication and Characterization

#### Composite Fabrication

Composites were fabricated via vacuum infiltration of the PEG hydrogel precursor solution into the pores of the SMP foam. Briefly, the hydrogel precursor solution was prepared in an Eppendorf tube. An SMP foam was immersed in the solution, and the tube was placed under vacuum for 3 min to facilitate penetration of the hydrogel solution into the pores. The solution-filled foam was then secured in a PolyJet 3D-printed VeroClear mold (Stratasys, Eden Prairie, Minnesota) within a 1.80 mm-diameter and 25.4 mm-length channel and placed in a furnace at 65 °C for 1 h to crosslink the hydrogel. Composites were lyophilized for characterization using the same process described for the bulk hydrogels.

#### Hydrogel Infiltration into SMP Foams

Hydrogel infiltration into the foam was evaluated by adding activated SAMSA fluorescein to the hydrogel precursor solution. SAMSA fluorescein was activated according to the manufacturer’s instructions. This thiol-containing fluorescein glows green under blue light (495 nm), allowing visualization of gel infiltration into the foam. 20 mm composites were made with the fluorescein-containing hydrogel solution, and thin slices were taken from the top, middle, and bottom sections. The faces of the slices were visualized with a fluorescence microscope (ZEISS Axio Vert A1). Intensity profiles along the diameter of the faces were generated with ImageJ.

### Shape Recovery

Control foams and fluorescein-containing composites (1.5–1.8 mm diameter) were measured via ImageJ, fixed along a nitinol wire, and crimped down to 0.8 mm diameter. Variability of the original diameter of samples was due to inconsistencies with the preparation of cylindrical SMP foams with a Dremel. The samples (*n* = 3) were placed in a 37 °C water bath. Composites were placed under UV light for visualization of the hydrogel during testing. Video and images were taken for analysis, and sample diameters were measured with ImageJ. Shape recovery was calculated according to eq 2.
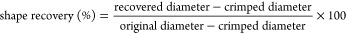
2

#### Cytocompatibility

The cytocompatibility of leachable byproducts from the composites were determined using an extract media test following ISO 10993-5 guidelines.^23^ L929s, mouse lung fibroblasts, were cultured with Minimum Essential Medium, 1% penicillin–streptomycin, 2 mM GlutaMAX, and 10% fetal bovine serum. Sample groups include a blank control (cell media), foams, unwashed composites, washed composites, and a cytotoxic control (*n* = 3). SMP foams were washed in a sonicator with three 15 min cycles in DI water and three 15 min cycles in isopropyl alcohol followed by drying in an oven at 80 °C overnight. Composites were washed in DI water for 4 h, changing the water after 2 h.

For testing, L929s were plated in a 24-well plate and grown to ∼80% confluency for 24 h. Meanwhile, the foams, unwashed composites, and washed composites were immersed in cell culture media at a concentration of 1 cm^2^/mL for 24 h. After 24 h, the cell culture media in the plate were replaced with the extract media. The plate was subsequently incubated at 37 °C and 5% CO_2_ for 24 h. An XTT assay was used to evaluate the cytotoxicity of the sample groups. Thirty minutes prior to evaluation, the media in the cytotoxic control group were replaced with 70% methanol. After 30 min, the methanol was removed and replaced with cell culture media. The XTT assay was used according to the manufacturer’s protocols (Biotium). The activated XTT solution was made by mixing the activation reagent with the XTT solution in a 1 μL:200 μL ratio. 500 μL of activated XTT solution was added to each well, and the plate was incubated at 37 °C and 5% CO_2_ for 19 h. Subsequently, the absorbances at 450 nm (sample) and 650 nm (background) were measured with a Cytation 5 plate reader (Agilent). Net absorbance was calculated by subtracting the background absorbance (*A*_650_) from the sample absorbance (*A*_450_). Percent viability for each sample was calculated via normalization to the mean of the blank control group.

### Benchtop Testing of Composites

#### Deployment Force

The forces required to deploy 10 and 20 mm-long composites were analyzed using a force gauge (DFS, Nextech Global). The deployment device consists of three components: a cannula needle, device housing, and pusher rod. Briefly, the 1.5 mm-diameter composites were crimped down to 0.8 mm and inserted into the device housing (PTFE tubing). Composites (*n* = 8) were soaked in water within the device housing for 30 s, 1 min, and 2 min. The composites were then deployed through the cannula needle against the force gauge using a pusher rod. The deployment force test is meant to portray the worst-case scenario—fluid working up into the device housing during deployment, causing premature actuation and swelling of the composite. A threshold of 5 N of force was set as the success criteria.

#### In Vitro Testing on the Lung Model

Control foams, 10 mm composites, and 20 mm composites (*n* = 8) were tested on an in vitro lung model to evaluate their sealing ability. The lung model consists of a wet silicone elastomer foam encased in a rigid plastic housing covered with tape to prevent air leakages. The housing unit is connected to a compressed air tank and immersed in a heated water bath. During testing, the housing unit is secured with a plastic lid that is connected to an air flow sensor to read air flow from the model via a LabView program. For testing, a needle is punched through the foam and tape to simulate a biopsy tract. The housing unit is then secured, and air flow is set to 40 mL/min. Once the air flow has stabilized at 40 mL/min for 1 min, water was added to the biopsy site followed by a biopsy tract sealant (control foams and composites). Sealants were placed into the tracts with the deployment device using a similar procedure for the placement of the BioSentry in vivo.^9^ For the in vitro model, the dry sealants were placed inside the device housing and connected to a marked cannula needle. The marked cannula needle was placed into the biopsy tract at a depth based on the length of the sealant (10 or 20 mm) so that the proximal face of the sealant is placed at the surface of the silicone foam (pleural) layer. A pusher rod was inserted to deploy the sealants, and the cannula needle and device housing were retracted. After sealant placement, the foam housing was secured and air flow was measured via the LabView program for 5 min. Results reported are the percent reduction of air flow, or the difference in the average air flow in the open biopsy tract (∼40 mL/min) and the average air flow in the plugged/treated biopsy tract divided by the average air flow in the open biopsy tract.

### Statistical Analysis

Data are shown as the mean ± standard deviation. One-way and two-way analyses of variance (ANOVA) followed by Tukey’s post hoc test were used to determine statistical significance of differences unless otherwise stated (ns *p* > 0.05, *p ≤ 0.05, **p ≤ 0.01, ***p ≤ 0.001, ****p ≤ 0.0001).

## Results and Discussion

### Hydrogel Synthesis and Characterization

**Figure 1:**
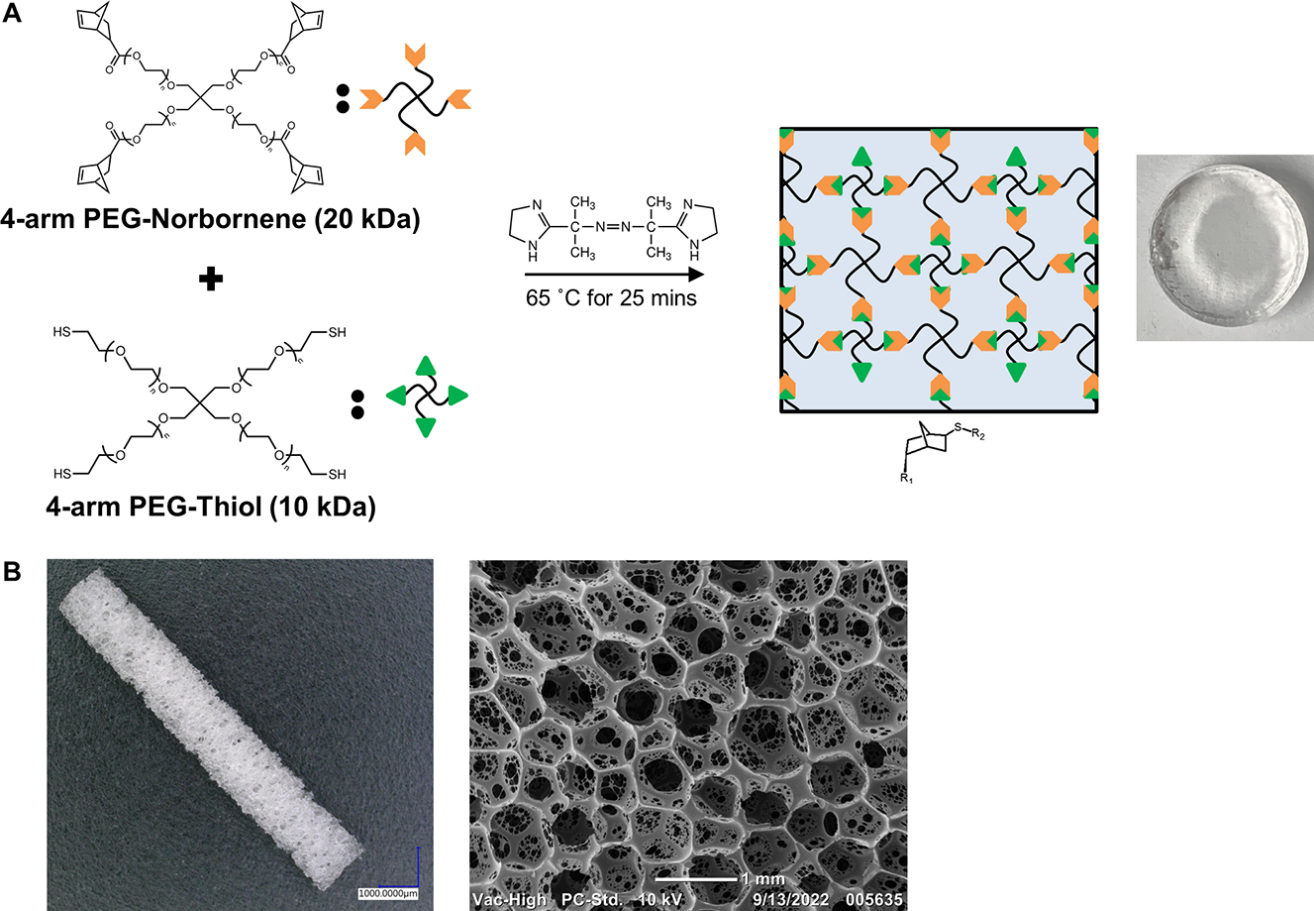
Materials for composite fabrication. (A) Thiol–ene crosslinking of the PEG hydrogel with a thermal azo initiator, VA-044. (B) Gross image and scanning electron micrograph of the polyurethane SMP foam.

The properties of the thiol–ene-crosslinked hydrogels at varying PEG wt % and thiol-to-ene ratios were characterized to select a formulation to be used with an SMP foam for composite fabrication (Figure 1). Swelling was of primary importance since the hydrogel will serve as a coating within the pores of the SMP foam and must absorb water and swell to fully seal the biopsy tract. However, other properties relevant to composite fabrication and performance were also characterized.

**Figure 2:**
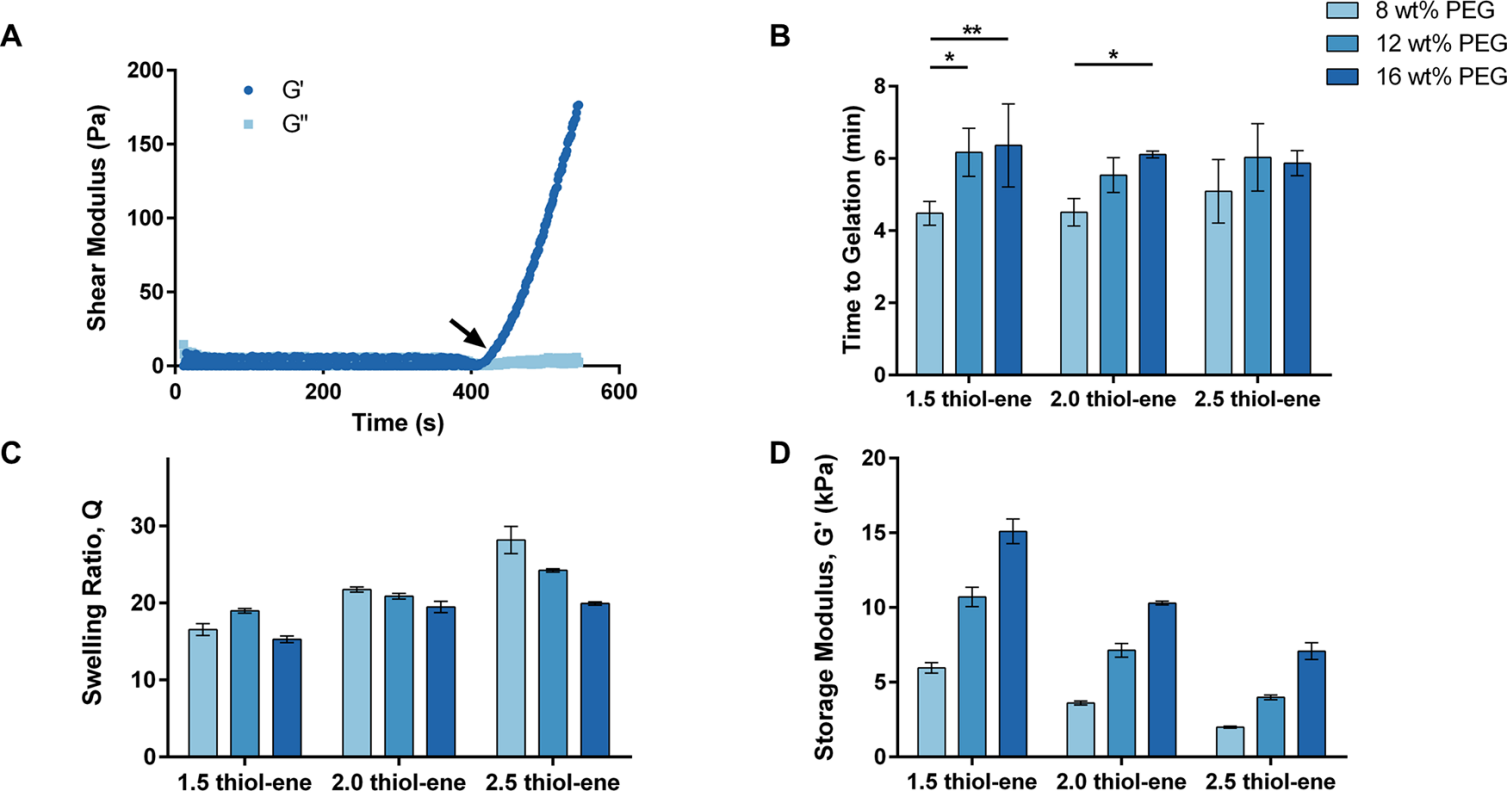
Characterization of PEG hydrogels at varying PEG concentrations and thiol–ene ratios. (A) Representative gelation curve of an 8 wt % and 2.5 thiol–ene hydrogel formulation. The arrow indicates the time to gelation (*G*′ = storage modulus and *G*″ = loss modulus). (B) Comparison of gelation time (*p ≤ 0.05, **p ≤ 0.01). (C) Comparison of the equilibrium swelling ratio at 1 rad/s and 1% shear strain. (D) Comparison of equilibrium storage modulus. *N* = 3 in (B–D).

Gelation kinetics studies were conducted to evaluate the amount of time required for curing during composite fabrication. A quick gelation time was desired to ensure minimal leakage of hydrogel precursor solution from the pores of the foams during composite fabrication. Figure 2A shows a characteristic gelation curve for an 8 wt % PEG and 2.5 thiol–ene ratio sample. A steep increase in *G*′ can be seen after ∼306 s, indicating the transition from solution to gel. There was a statistically significant increase from 8 wt % (4.48 ± 0.33 min) to 12 wt % (6.17 ± 0.66 min) and 16 wt % (6.36 ± 1.15 min) for the 1.5 thiol–ene formulation and from 8 wt % (4.51 ± 0.38 min) to 16 wt % (6.11 ± 0.09 min) for the 2.0 thiol–ene formulation (Figure 2B). Other differences were not statistically significant. While slower gelation at higher PEG concentrations was unexpected, this trend can be attributed to heat absorption by the polymer. Since solutions with higher concentrations of PEG will absorb more heat, it will take longer for the hydrogel solution to reach the target temperature, increasing the time to gelation. Regardless, all formulations gelled in 4.5–6.5 min, which was sufficient for composite fabrication.

Equilibrium swelling ratios were measured to evaluate the capacity of each formulation to absorb water. Figure 2C compares the equilibrium swelling ratios of hydrogel disks with varying PEG wt % and thiol–ene ratios after swelling in PBS for 17 h. In general, the swelling ratios decreased as the content of PEG increased, with the swelling ratios for the 8, 12, and 16 wt % PEG and 2.5 thiol–ene formulations significantly decreasing (*p* ≤ 0.0001) with increasing PEG concentration (28.2 ± 1.8, 24.2 ± 0.2, 19.9 ± 0.2, respectively). This trend can be attributed to the increase in crosslink density associated with the increase in PEG content. Furthermore, an increase in the thiol–ene ratio increased the equilibrium swelling ratio. There was a statistically significant increase (*p* ≤ 0.0001) as the thiol–ene ratio was increased from 1.5, 2.0, and 2.5 for an 8 wt % PEG formulation (16.6 ± 0.8, 21.7 ± 0.3, and 28.2 ± 1.8, respectively). This effect was due to the increase in the molecular weight between crosslinks and, thus, decrease in crosslink density. These trends were as expected. Overall, all formulations had swelling ratios indicative of a high capacity for water absorption.

The mechanical properties of the hydrogels were also evaluated by measuring their equilibrium storage moduli (*G*′) after swelling in PBS for 17 h. Figure 2D shows the storage moduli for the hydrogel disks, measured at a frequency of 1 rad/s and 1% shear strain. The storage modulus increased significantly with increasing PEG content and decreased significantly with increasing thiol–ene ratio. The 8, 12, and 16 wt % PEG (2.5 thiol–ene ratio) formulations had an equilibrium storage modulus of 1.99 ± 0.06, 3.99 ± 0.16, and 7.08 ± 0.55 kPa, respectively. The 1.5, 2.0, and 2.5 thiol–ene ratio (8 wt % PEG) formulations had an equilibrium storage modulus of 5.96 ± 0.36, 3.61 ± 0.14, and 1.99 ± 0.06 kPa, respectively. All differences between sample groups were statistically significant (*p* ≤ 0.001). As with the equilibrium swelling ratio results, these trends were due to the effects of PEG content and thiol–ene ratio on the crosslink density of the hydrogel formulations.

**Figure 3:**
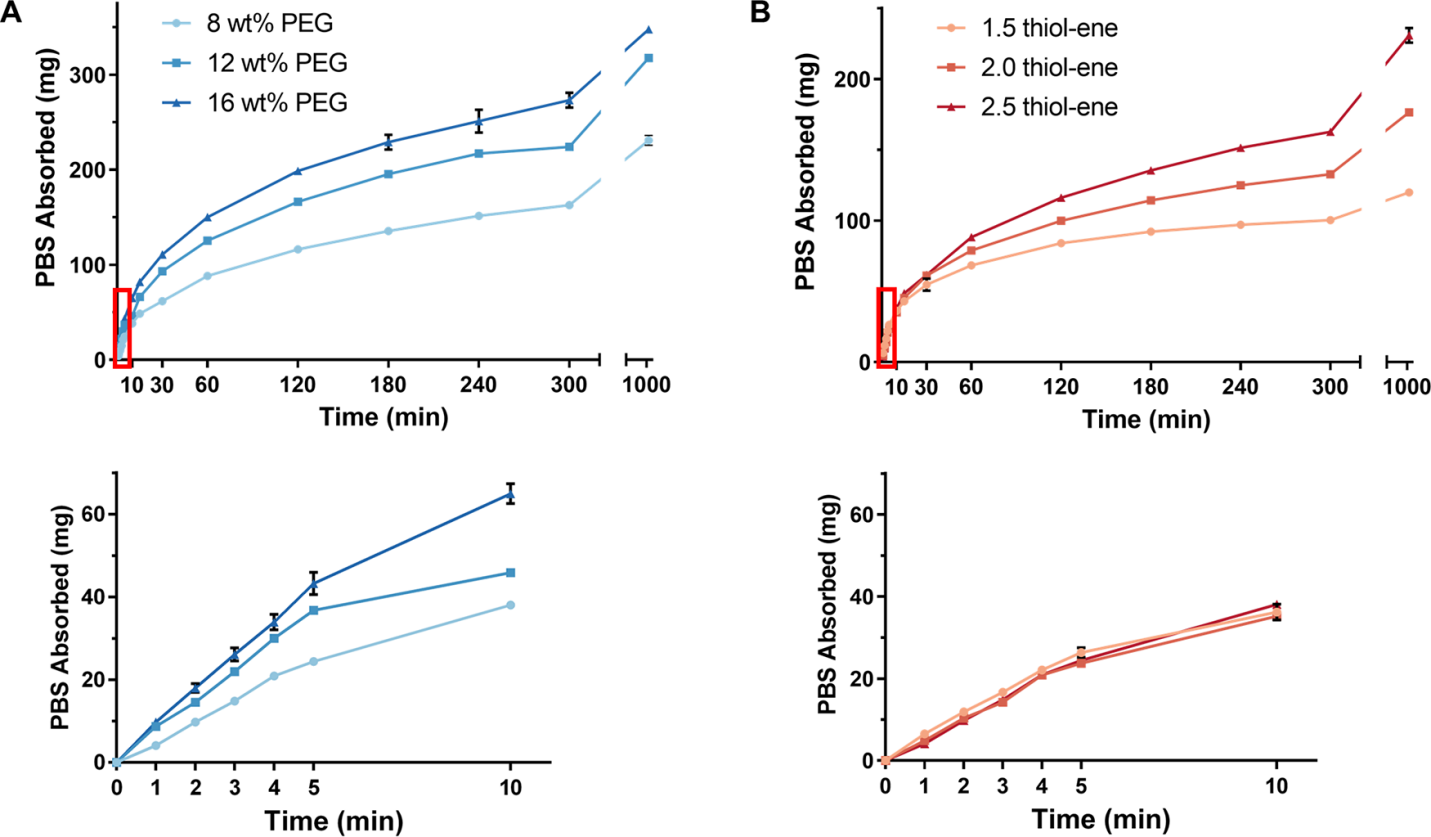
Swelling kinetics of desiccated PEG hydrogels. The red rectangle is the area represented in the bottom graphs. (A) Comparison of swelling kinetics at different PEG concentrations and 2.5 thiol–ene ratio. (B) Comparison of swelling kinetics at different thiol–ene ratios and 8 wt % PEG. *N* = 3 in all panels.

**Figure 4:**
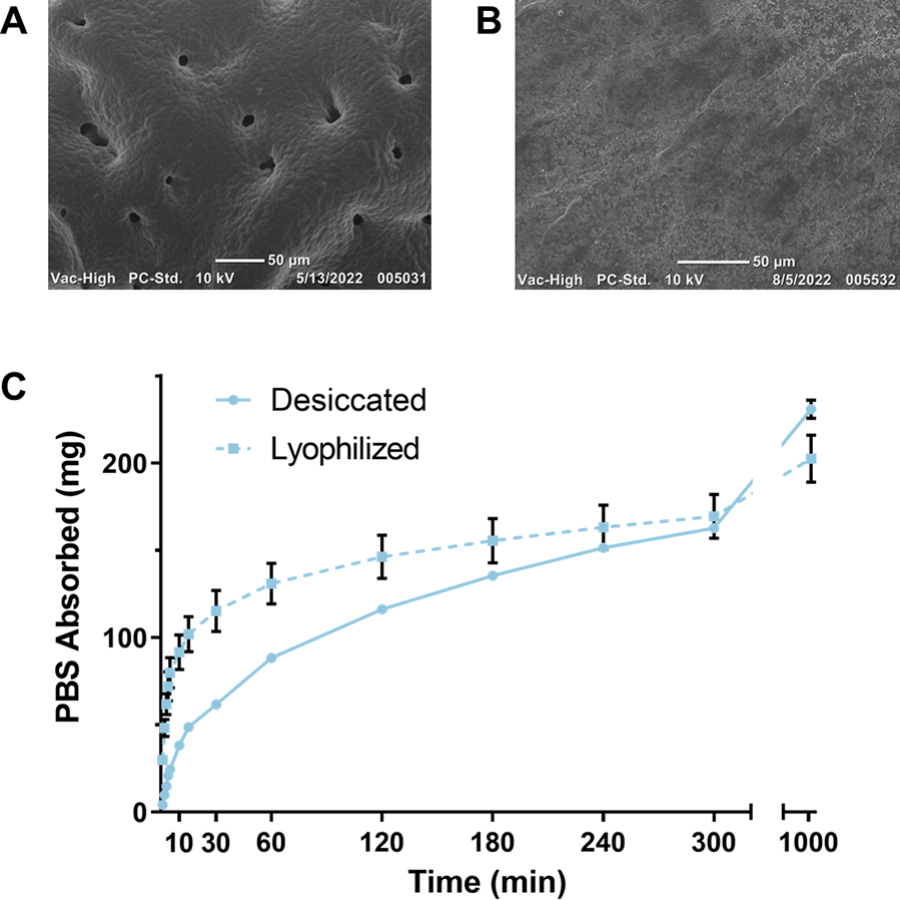
Comparison of lyophilized and desiccated PEG hydrogels. (A) Scanning electron micrograph of a lyophilized hydrogel. (B) Scanning electron micrograph of a desiccated PEG hydrogel. (C) Comparison of the swelling kinetics of a lyophilized and desiccated 8 wt % PEG and 2.5 thiol–ene ratio hydrogel (*n* = 3).

Understanding the water absorption kinetics of the hydrogel formulations is critical to the application, and it is important that the hydrogels swell quickly to seal the biopsy tract. Thus, the swelling kinetics were studied by drying the hydrogels and measuring the mass of PBS absorbed and change in diameter over time. PBS absorption and diameter for a 2.5 thiol–ene hydrogel increased with increasing PEG content over the entire test (Figure 3A, Figure S2A). For instance, at 10 min, the 8, 12, and 16 wt % PEG formulations had absorbed 38.1 ± 1.0, 45.9 ± 0.5, and 64.9 ± 2.4 mg of PBS, respectively. When increasing the thiol–ene ratio (for an 8 wt % PEG hydrogel), the mass of PBS absorbed and diameter measurements were similar at the early timepoints but then began to increase after 60 min (Figure 3B, Figure S2B). The 1.5, 2.0, and 2.5 thiol–ene formulations had absorbed 36.2 ± 1.9, 35.2 ± 0.6, and 38.1 ± 1.0 mg of PBS at 10 min and 68.3 ± 2.3, 78.8 ± 1.3, and 88.3 ± 1.5 mg of PBS at 60 min. Collectively, these results show that PEG content is the primary driver for PBS uptake due to the increase in hydrophilicity of the network. The effects of lyophilization on the swelling kinetics were also studied for the 8 wt % PEG and 2.5 thiol–ene ratio hydrogel formulation to determine if lyophilization can further increase the rate of water absorption. Figure 4C shows the comparison of PBS uptake of a lyophilized hydrogel versus a desiccated hydrogel. The lyophilized gels swelled much faster than the desiccated gels due to the creation of pores within the lyophilized gel, shown by the SEM image in Figure 4A. Lyophilization resulted in an increase in the swelling rate from 5.1 ± 0.2 to 15.4 ± 1.9 mg/min (Table 1). The difference between the rates was statistically significant (*p* ≤ 0.001), according to an unpaired *t*-test.

**Table 1:** Rate of PBS Absorption for Each Sample Group over the First 5 Min in PBS (*N* = 3)^a^

group	PEG wt %	SH:Ene	desiccated or lyophilized	swelling rate (mg/min)
1	8	1.5	desiccated	5.23 ± 0.21
2	8	2	desiccated	4.86 ± 0.13
3	8	2.5	desiccated	5.08 ± 0.19
4	12	1.5	desiccated	5.96 ± 0.03
5	12	2	desiccated	7.44 ± 0.21^***,####^
6	12	2.5	desiccated	7.30 ± 0.08^***,####^
7	16	1.5	desiccated	7.76 ± 0.16^####^
8	16	2	desiccated	8.98 ± 0.48^***,####^
9	16	2.5	desiccated	8.48 ± 0.53^####^
10	8	2.5	lyophilized	15.43 ± 1.93^†††^

a * indicates significance between comparisons at the same PEG weight percent and different thiol-to-ene ratios. # indicates significance between comparisons at the same thiol-to-ene ratio and different PEG weight percent. † indicates significance between lyophilized and desiccated 8 wt % PEG and 2.5 thiol-to-ene hydrogels.

### Composite Fabrication and Characterization

**Figure 5:**
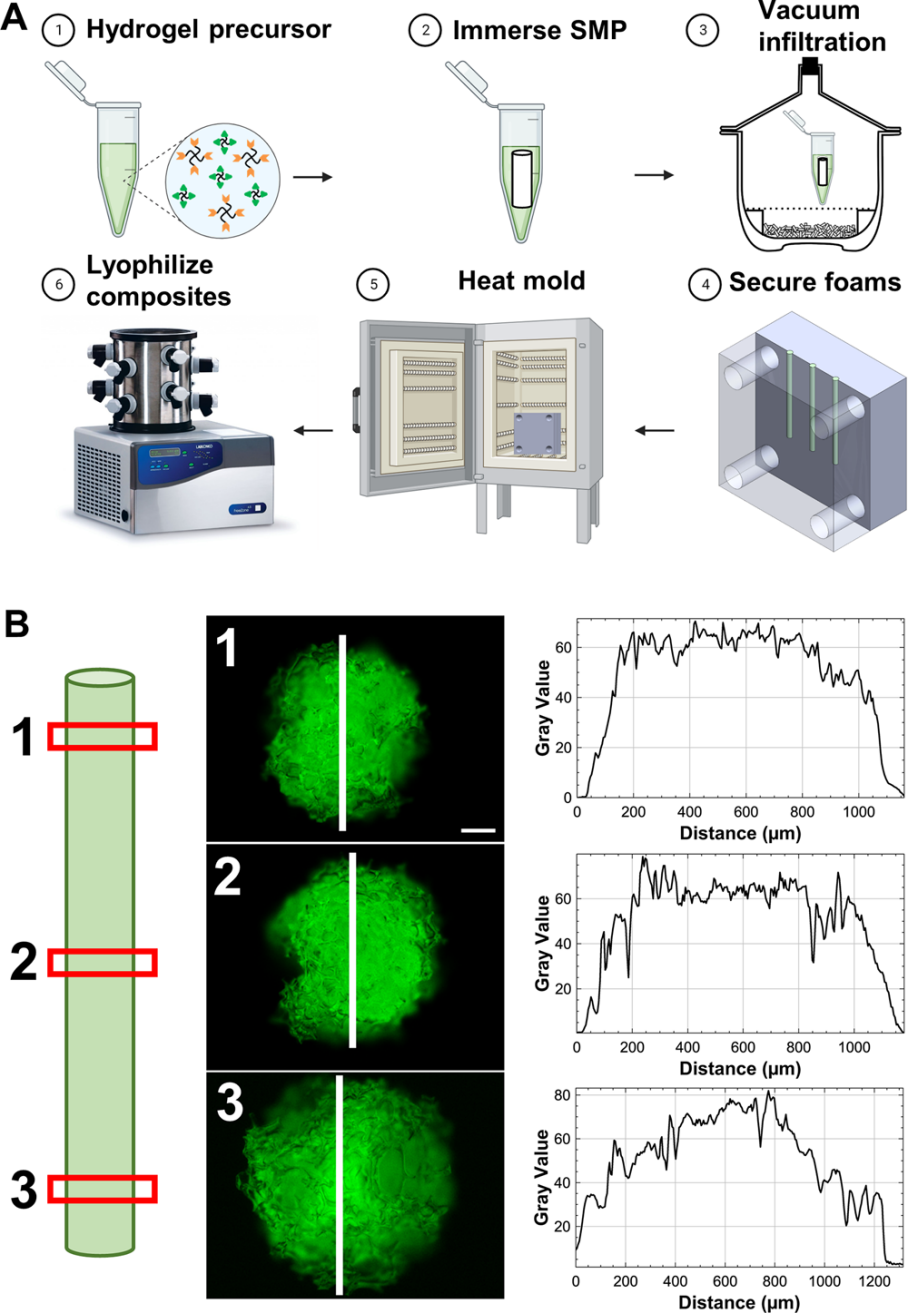
(A) Process overview for composite fabrication. (B) Evaluation of hydrogel incorporation in the composites. Fluorescein was incorporated in the hydrogel for visualization. Red boxes indicate the locations of the cross sections visualized in the representative fluorescent micrographs (scale bar: 200 μm). Intensity profiles on the right were generated along the white lines.

Composites were produced using the 8 wt % PEG and 2.5 thiol–ene ratio hydrogel formulation and a 1.5 mm-diameter SMP foam (10 and 20 mm in length). Figure 5A shows a detailed outline of the composite fabrication method. Ten and 20 mm SMP foams were immersed in 50 and 80 μL of hydrogel precursor solution, respectively. The 3D-printed mold effectively secured the SMP foam and reduced leakage of hydrogel solution during crosslinking. Furthermore, it disassembled along the long axis of the composites, allowing for their easy removal post-crosslinking.

To evaluate the effectiveness of the composite fabrication method, the hydrogel infiltration into the SMP foam was assessed with SAMSA fluorescein. The thiol-containing fluorophore was covalently incorporated into the hydrogel network during crosslinking and enabled visualization of the hydrogel by fluorescence microscopy. Figure 5B shows the location of slices (red rectangles) taken from a 20 mm composite for analysis. The faces of each slice are depicted along with the region of interest (white line) and corresponding intensity profile (Figure 5B). Notably, the images show hydrogel throughout the entire face of each slice and the intensity profiles had high gray values in the middle of each face, indicating effective infiltration. This data demonstrates that our fabrication technique results in a composite with hydrogel throughout the entire foam, rather than just a coating on the exterior.

**Figure 6:**
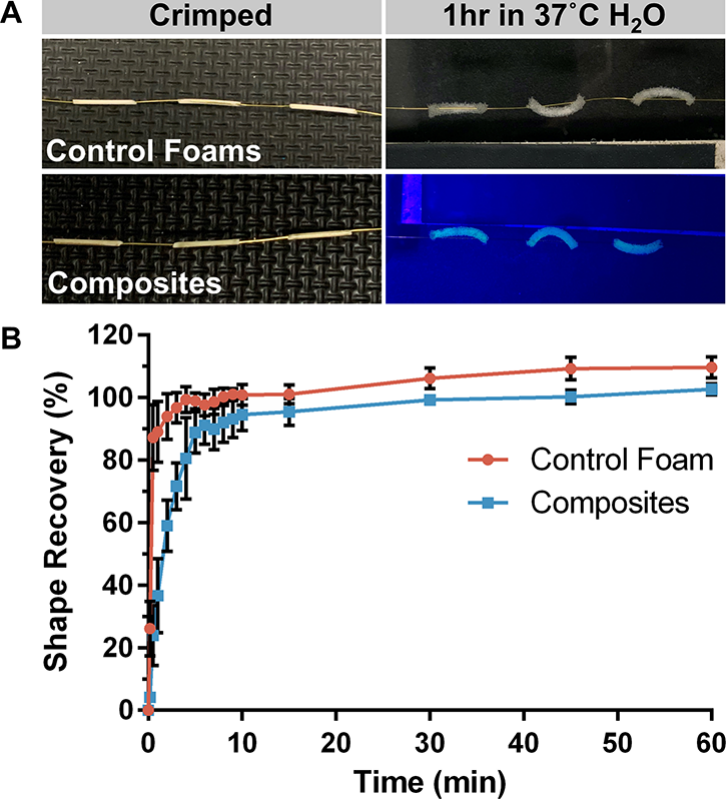
Characterization of composite shape recovery. (A) Representative images of control foams (top) and composites (bottom) during testing at *t* = 0 (crimped) and *t* = 1 h. (B) Comparison of % shape recovery of control foams and composites. Plot shows average percentage ± standard deviation (*n* = 3).

Next, the effects of hydrogel incorporation on the shape recovery of the composites were compared to control SMP foams. As has previously been shown for polyurethane SMPs, the dry glass transition temperature (*T*_g_) of the SMP foams was much higher than room temperature (48.8 °C ± 5.7 °C; Figure S3). Thus, plasticization by water is required for actuation. Figure 6 shows the experimental setup and results. The top samples are the control foams, and the bottom samples are the fluorescein-containing composites (Figure 6A). The control foams expanded rapidly, reaching 87.29% shape recovery within 30 s of being in the 37 °C water bath. In contrast, the composites reached 80.54% shape recovery in 4 min (Figure 6B). Thus, the hydrogel had a minor effect on the expansion and shape recovery of the SMP foam due to the hydrogel slowing water uptake by the foam, which is necessary for actuation. However, we expect that this rate of expansion is sufficient for sealing of the biopsy tract since the final diameter of the composite (∼1.5 mm) is much larger than the size of a biopsy tract (∼0.9 mm, a 20 G needle). Furthermore, this delayed expansion may be advantageous for the working time of our sealant—a problem with the BioSentry Lung Biopsy Sealant.

**Figure 7:**
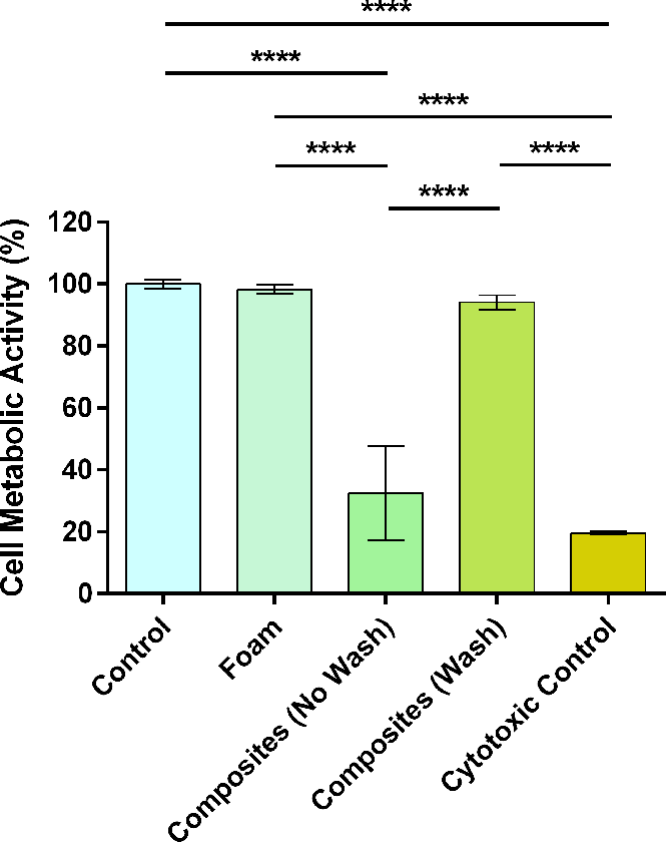
Cytocompatibility of leachable byproducts from control (no material), foams, unwashed composites, washed composites, and cytotoxic control (70% methanol). *N* = 3: ****p ≤ 0.0001.

An extract media test was performed to assess potential leaching of cytotoxic byproducts, and the effects of washing the composites were evaluated. The SMP foams, unwashed composites, washed composites, and cytotoxic control had cell metabolic activities of 98.2 ± 1.3, 32.5 ± 12.4, 94.1 ± 1.9, and 19.6 ± 0.5%, respectively (Figure 7). Importantly, there were no significant differences between the control, foam, and washed composite groups. However, the unwashed composite group was significantly lower compared to the foam and washed composite groups (*p* ≤ 0.0001). These results demonstrate that the addition of the hydrogel introduces cytotoxic leachables, which can be removed via a simple washing process—4 h in DI water, changing the water after 2 h. Although we did not analyze the cytotoxic leachables from the unwashed composites, they are most likely the unreacted VA-044 initiator, which has been found to be cytotoxic at 0.125 w/v% and above.^24^ The VA-044 concentration used for composite fabrication was 0.5 wt %.

### Benchtop Testing of Composites

**Figure 8:**
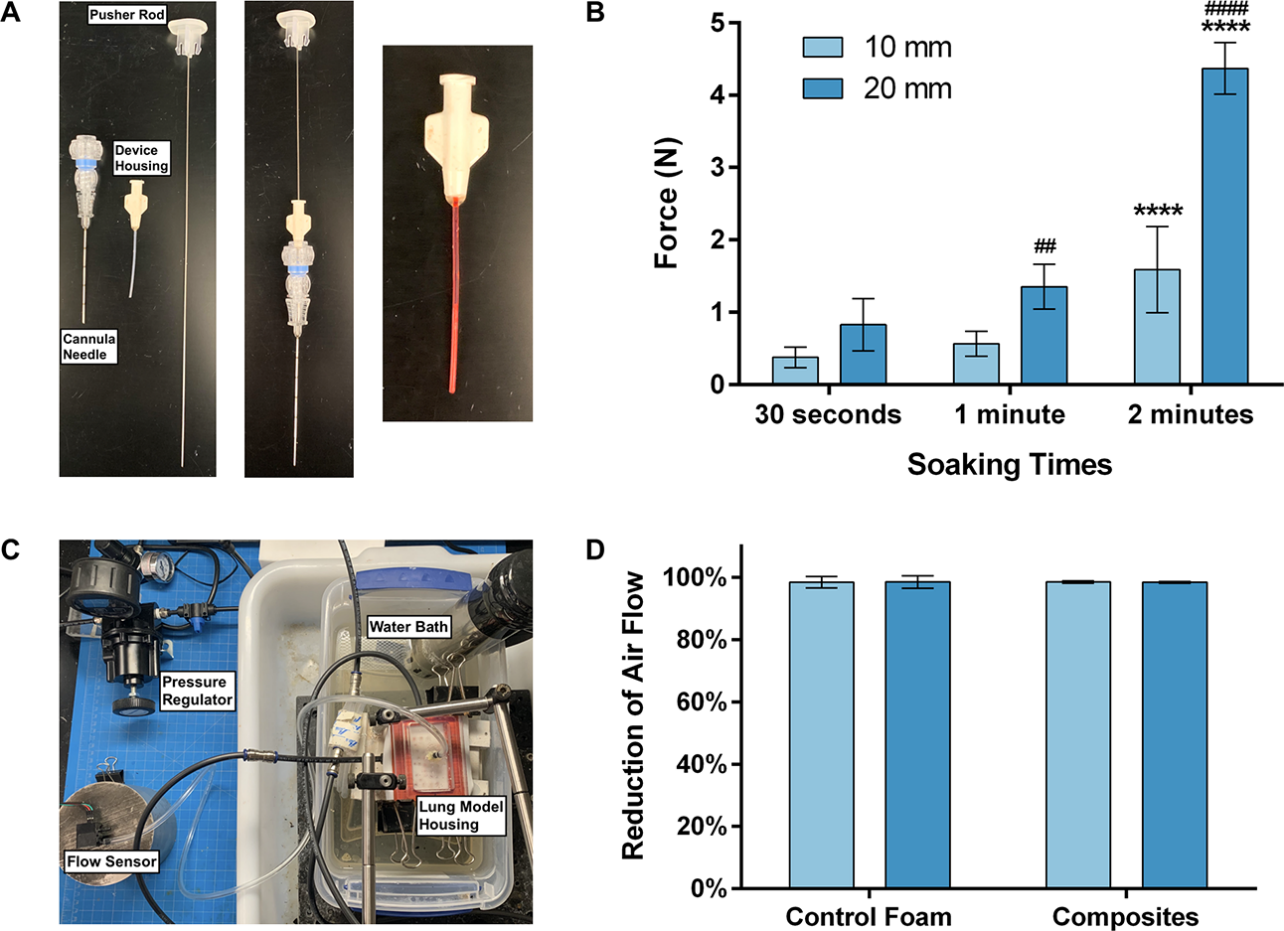
Benchtop characterization of composites. (A) Exploded view (left) and connected view (middle) of delivery device components and image of composite soaking within the device housing (right). (B) Deployment force measurements after 30 s, 1 min, and 2 min for 10 and 20 mm composites. * indicates significance between comparisons at the same length and different soaking times. # indicates significance between comparisons at the same soaking time and different lengths. (C) In vitro lung model apparatus for testing of sealing efficacy. (D) Comparison of lung biopsy sealing ability of 10 and 20 mm control foams and composites. Percent reduction from air flow in the open biopsy tract (∼40 mL/min). *N* = 8 in (B, D).

Deployment force testing was done for a possible “worst-case” scenario of fluid from the biopsy, working its way into the device housing, prematurely actuating and swelling the composite. Exploded and connected views of the components of the overall biopsy sealant device system are shown in Figure 8A. The components are as follows: a cannula needle, the device housing, and a pusher rod. The final image in Figure 8A shows a composite soaking in red water within the device housing. For the 10 mm composites, the force required increased as the soaking time increased with 30 s, 1 min, and 2 min of soaking time requiring 0.38 ± 0.14, 0.57 ± 0.17, and 1.59 ± 0.60 N of force (Figure 8B). However, while the force required increased, all samples were successfully deployed under 5 N of force, which is a low threshold sufficient for handheld use. The 20 mm composites were successfully deployed after 30 s and 1 min of soaking, requiring 0.83 ± 0.36 and 1.35 ± 0.31 N of force, respectively. However, only three samples (mean deployment force of 4.37 ± 0.36 N shown in Figure 8B) were deployed after 2 min. The other five samples buckled within the device housing and could not be deployed. Thus, if fluid enters the device housing, it is critical that a 20 mm composite be deployed within 1–1.5 min. However, dry composites can be easily deployed without any appreciable force.

Finally, a lung model was used to evaluate the efficacy of the composites for sealing lung biopsy tracts (Figure 8C). The lung model was composed of a silicone elastomer foam encased in a hard housing unit connected to a compressed air tank, and a tract was punched through the “lung” with a cannula needle to simulate a lung biopsy. Ten and 20 mm control foams and composites were placed into the tract and tested on the in vitro model. After setting the air flow to 40 mL/min, the samples were inserted, the model was secured, and the air flow was measured for five minutes (Figure S4). An air flow leak rate of 40 mL/min was used to simulate a leak rate that would require medical intervention, like the placement of a chest tube, and be susceptible to recurrent air leaks.^25,26^Figure 8D shows the percent reduction of air flow after treatment with each group. The composites (10 and 20 mm) reduced the air flow by 98.48% ± 0.42% and 98.36% ± 0.23%, respectively, indicating the effective sealing of the biopsy tract. Thus, these results demonstrate that a 10 or 20 mm composite has the potential to serve as a lung biopsy sealant device. Although the control foams were also able to seal the tract, the addition of the hydrogel allows for increased functionality of the device. For example, the hydrogel can be functionalized with antimicrobial agents or loaded with a drug to promote healing of the biopsy tract.^27,28^ While degradation of the materials was not studied, both components of the composites are expected to degrade in vivo. Specifically, the PEG hydrogel component will degrade primarily by hydrolysis due to the labile ester bonds in the PEG-Norbornene precursor, whereas the polyurethane SMP foam will degrade oxidatively via cellular activity and free radicals in the tissue.^29^

## Conclusions

This study reports the successful development of PEG hydrogel/SMP foam composites and demonstrates their potential to serve as a lung biopsy sealant. Characterization of the composites revealed uniform hydrogel infiltration throughout the SMP foam. While the addition of hydrogel had a small effect on the shape recovery of the SMP foam, the composites recovered their shape within 4 min. This slight delay in shape recovery can be beneficial for the application by increasing the working time. The composites were not cytotoxic to L929s after a 4 h wash in DI water. Furthermore, the composites were deployed after soaking within the device housing for up to 2 min and were effective in sealing a lung biopsy tract in an in vitro lung model. Future studies will evaluate the efficacy of these composites in vivo.

## Supporting Information Available

NMR spectra of PEG-NB for calculation of percent functionalization, change in diameter of desiccated PEG hydrogels with varying thiol–ene ratios and PEG wt % during swelling, DSC curves of polyurethane SMP foams, and example plot of the in vitro sealing data (PDF)
