# Supplementary material for: Swellable and Thermally Responsive Hydrogel/Shape Memory Polymer Foam Composites for Sealing Lung Biopsy Tracts
Source: ACS Biomater Sci Eng. 2023 Feb 2;9(2):642–50. doi: 10.1021/acsbiomaterials.2c01369 (PMC10726527; doi:10.1021/acsbiomaterials.2c01369)
Supplement: Supplementary file 1 — ab2c01369_si_001.pdf [file ab2c01369_si_001.pdf]

SUPPORTING INFORMATION

Swellable and Thermally Responsive Hydrogel/Shape Memory Polymer Foam Composites for Sealing Lung Biopsy Tracts

*Matthew A. Jungmann[1], Sarea Recalde Phillips[1], Tyler J. Touchet[1], Braeden Brinson[1], Katherine Parish[2], Corinne Petersen[1], Sayyeda Marziya Hasan[3], Landon D. Nash[3], Duncan J. Maitland[1,3], Daniel L. Alge[1,4]**

[1]Department of Biomedical Engineering, Texas A&M University, College Station, TX 77843

[2]Department of Chemical Engineering, Texas A&M University, College Station, TX 77843

[3]Shape Memory Medical, Inc., Santa Clara, CA 95054, USA

[4]Department of Materials Science & Engineering, Texas A&M University, College Station, TX 77843

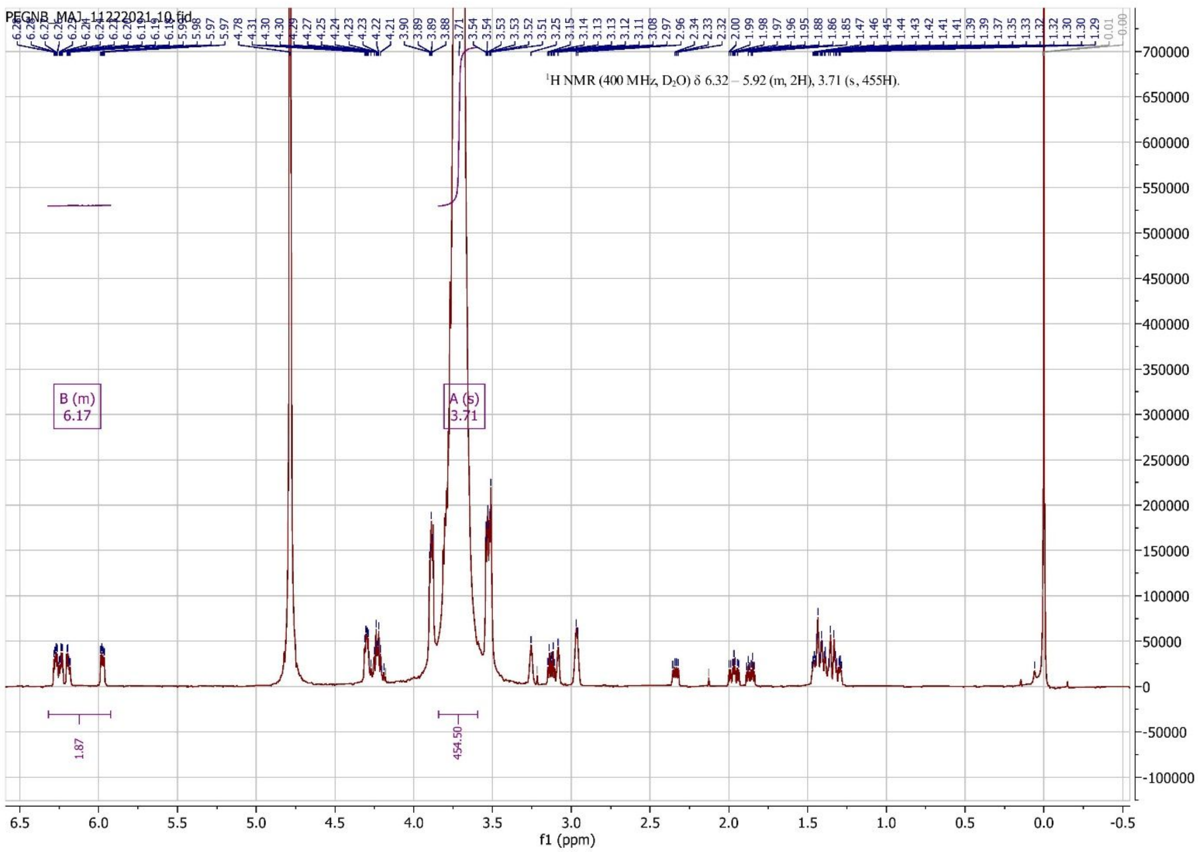

Figure S1. NMR spectra of 4-arm 20 kDa PEG-NB. A represents the hydrogens associated with the poly(ethylene glycol) chain on one arm (~454.50). B represents the normalized number of hydrogens associated with the alkene on the norbornene of one arm (1.87). Since the number of hydrogens on the alkene of the norbornene should be 2, we can calculate the % functionalization of our PEG-NB by taking (1.87/2) × 100 which is a 93.5% functionalization.

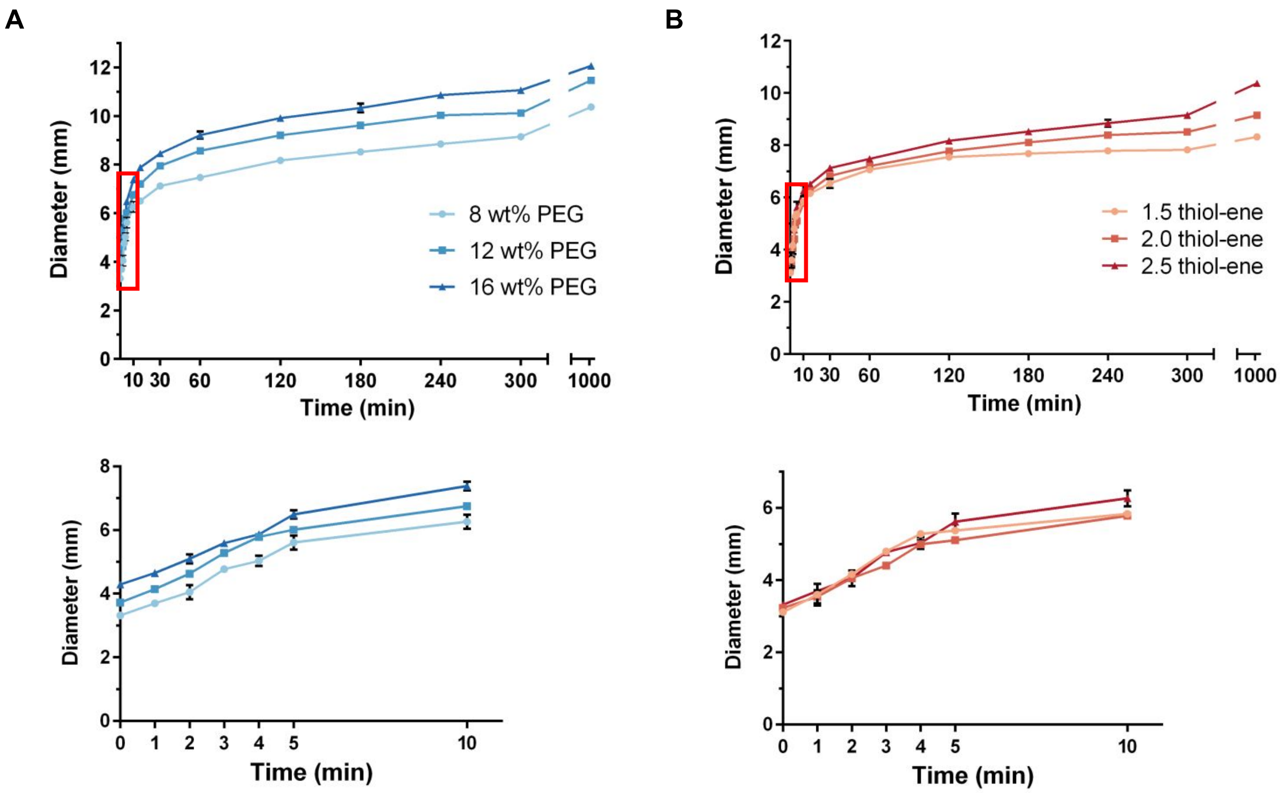

Figure S2. Change in diameter of desiccated PEG hydrogels during swelling. The red rectangle is the area represented in the bottom graphs. (A) Comparison of diameter at different PEG concentrations and 2.5 thiol-ene ratio. (B) Comparison of diameter at different thiol-ene ratios and 8 wt% PEG.

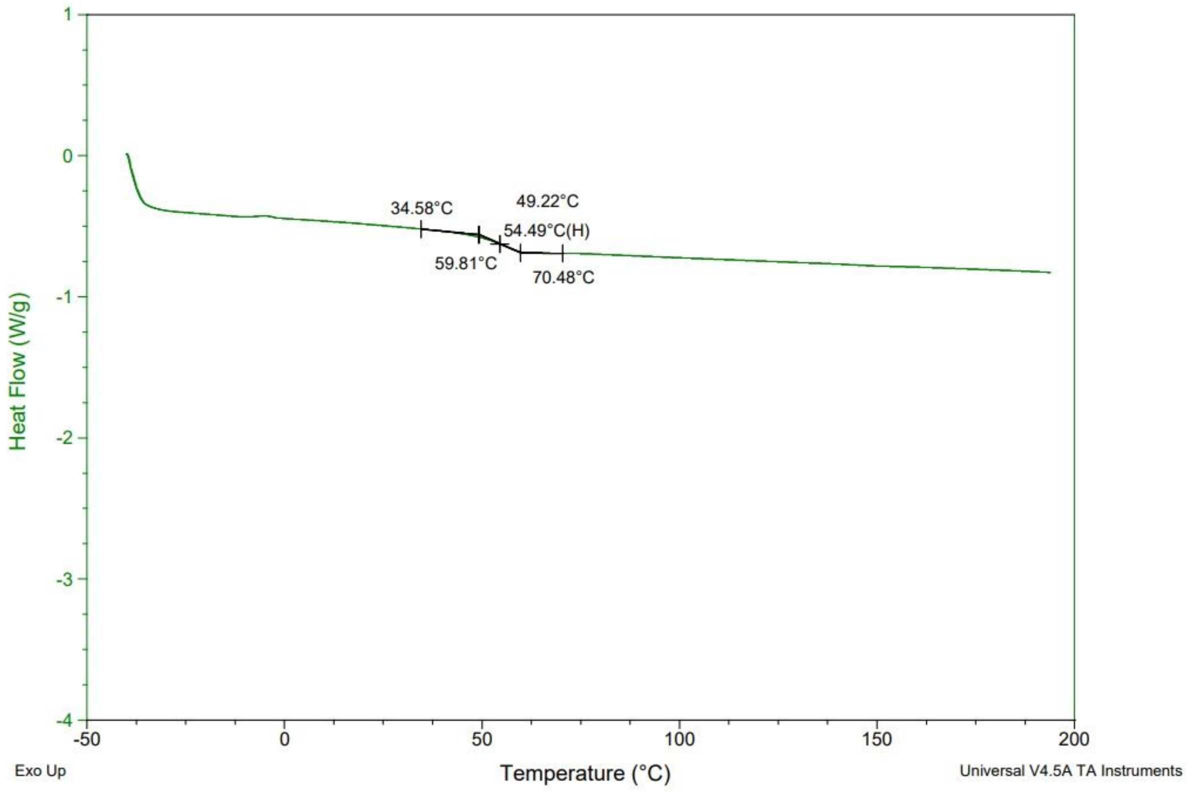

Figure S3. Representative DSC curve of the polyurethane SMP foams. Glass transition temperatures (T_g) is marked with the 'plus' sign (value marked with H).

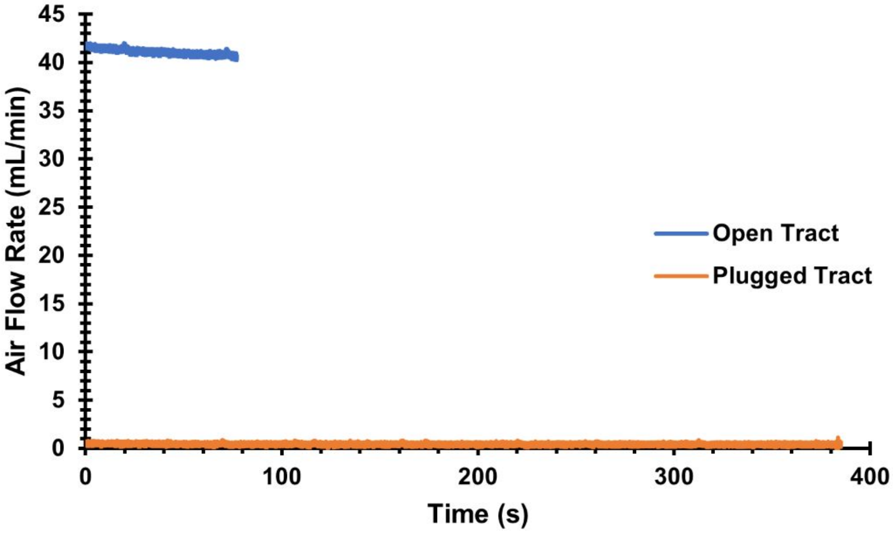

Figure S4. Representative plot of the *in vitro* sealing data on a 10 mm composite sample.
